# The Disabilities of the Arm, Shoulder and Hand Questionnaire (DASH) can measure the impairment, activity limitations and participation restriction constructs from the International Classification of Functioning, Disability and Health (ICF)

**DOI:** 10.1186/1471-2474-9-114

**Published:** 2008-08-20

**Authors:** Diane Dixon, Marie Johnston, Margaret McQueen, Charles Court-Brown

**Affiliations:** 1 Department of Psychology, University of Stirling, Stirling, Scotland, UK; 2 Institute of Applied Health Sciences, University of Aberdeen, Aberdeen, Scotland, UK; 3 Edinburgh Orthopaedic Trauma Unit, Royal Infirmary of Edinburgh, Edinburgh, Scotland, UK

## Abstract

**Background:**

The International Classification of Functioning, Disability and Health (ICF) model of the consequences of disease identifies three health outcomes, impairment, activity limitations and participation restrictions. However, few orthopaedic health outcome measures were developed with reference to the ICF. This study examined the ability of a valid and frequently used measure of upper limb function, namely the Disabilities of the Arm, Shoulder and Hand Questionnaire (DASH), to operationalise the ICF.

**Methods:**

Twenty-four judges used the method of Discriminant Content Validation to allocate the 38 items of the DASH to the theoretical definition of one or more ICF outcome. One-sample *t*-tests classified each item as measuring, impairment, activity limitations, participation restrictions, or a combination thereof.

**Results:**

The DASH contains items able to measure each of the three ICF outcomes with discriminant validity. The DASH contains five pure impairment items, 19 pure activity limitations items and three participation restriction items. In addition, seven items measured both activity limitations and participation restrictions.

**Conclusion:**

The DASH can measure the three health outcomes identified by the ICF. Consequently the DASH could be used to examine the impact of trauma and subsequent interventions on each health outcome in the absence of measurement confound.

## Background

Fracture of the wrist is a common injury and is especially prevalent in older post-menopausal women. Wrist fracture is associated with variable outcome, for example, approximately 50% of cases fail to recover pre-operative function [1] and 20% of patients report moderate to severe pain at 6 month post-fracture [2]. A recent series of Cochrane reviews of the effectiveness of commonly applied interventions for wrist fracture have highlighted the continued unsatisfactory outcome for many patients [3]. Further, there is an association between wrist fracture and long-term disability and increased risk of dependency [4]. Any measure of outcome from wrist fracture needs to be able to index the impact of fracture, not only on the impairment itself but also on the level of associated disability.

The WHO identifies three health outcomes in its International Classification of Functioning Disability and Health (ICF) taxonomy of the consequences of disease, namely, impairments (I), activity limitations (A) and restrictions in social participation (P) [5] (see Table 1 for definitions). In accordance with the ICF, a complete assessment of outcome for any health condition or intervention requires an evaluation of each health outcome domain. Whilst, the reliability and construct validity of six patient self-report instruments for the assessment of the upper extremity following trauma were recently reviewed [6], the review did not examine the content validity of each instrument relative to the outcomes identified in the ICF. This may be an important omission because the application of the ICF to aid rehabilitation requires that each health outcome be measurable. Whilst new measures specifically designed to assess each ICF outcome could be developed, the development and validation of new measures is a time consuming and expensive process. A preferable approach might be to examine the compatibility between the ICF and outcome measures currently in use [7].

**Table 1:** Definitions of the three constructs from the ICF Model [5]

Variable	Definition
Impairments (I)	Problems in body function or structures such as significant deviation or loss
	Body Functions are the physiological functions of the body systems (including psychological functions) Body Structures are anatomical parts of the body such as organs, limbs and their components

Activity limitations (A)	Difficulties an individual may have in executing activities
	Activity is the execution of a task or action by an individual

Participation restrictions (P)	Problems an individual may experience in involvement in life situations
	Participation is the involvement in a life situation

The development of core measurement sets for patients with musculoskeletal conditions or for acute hospital and early post-acute rehabilitation are available [8-10], and these may be relevant to patients with wrist fracture. Whilst, the core measurement sets detail the categories within each health outcome that need to be assessed for a given health condition, they do not identify the actual items to be used to measure each category. The ability of existing health outcome measures to operationalise the ICF is an area of active research [11-13] and it is possible that existing wrist fracture outcome measures may contain items that measure each ICF outcome. That said, in order for the ICF to be tested as a model of the outcomes from wrist fracture it is not only necessary to have measures of each ICF outcome but those measures need to show discriminant validity. Such pure measures are necessary to ensure that any observed relationship between the three outcomes is not simply due to measurement confound.

The method of Discriminant Content Validity (DCV) is able to establish the discriminant validity of measurement items [13,14]. DCV examines the relationship between individual measurement items and all constructs within a theoretical model thereby establishing the content validity of a measurement item against all constructs within a given theory. Rather than judge items against a single theoretical construct, the DCV method establishes whether each theoretical construct can be measured discriminately because the method asks judges to indicate the extent to which an item matches each theoretical construct of interest, in this case the three main outcomes identified by the ICF. This method has been used to establish the content validity of items within existing orthopaedic and chronic pain measures [13,14]. In this study we used DCV to identify ICF outcomes measured by the Disabilities of the Arm, Shoulder and Hand Questionnaire (DASH) [15].

## Method

### Design

Participants acted as judges and matched the thirty-eight items from the DASH to the definitions of the impairment, activity limitations and participation restrictions constructs from the ICF model.

### Participants

Twenty-four academics (one clinical, five industrial and thirteen health psychologists and five health service researchers) from the Applied Psychology Research Group at the University of Aberdeen took part in the study as part of a seminar on the DCV method. The precise number of judges required for judgement tasks is yet to be established but between 2–20 is regarded as adequate [16-18].

### Materials

The definitions of the three ICF constructs, namely: impairment, activity limitations, and participation restrictions were taken from the WHO and are given in Table 1. All 38-measurement items from the DASH were assessed. The DASH is a self-administered region-specific outcome instrument developed as a measure of self-rated upper-extremity disability and symptoms and has been identified as the most validated and easy to use measure of upper extremity function [6]. The DASH consists of 30 core items, and 8 optional items, which generate a disability score, scaled 0 (no disability) to 100.

### Procedure

The detailed procedure for a DCV study has been published previously [13,14,19]. Briefly, for each DASH item, participants provided a Yes/No judgement of whether the item was a match to the theoretical definition of each ICF construct. Consequently, each participant provided 3 judgements for each of the 38 items, i.e. 114 judgements in total. In addition, participants gave a confidence rating for each judgement on an 11-point scale ranging from 0% to 100%, rising in 10% increments.

### Statistical Analysis

#### Classification of items

Judgements were coded 1 for a match and -1 for a no match. Each judgement was multiplied by its accompanying confidence rating, expressed as a proportion. Consequently, the weighted judgements ranged from -1 to +1. One-sample t-tests were used to classify each item to one of the 7 possible combination of constructs, namely: I, A, P, IA, IP, AP or IAP. An item was classified as being related to a construct if its weighted judgement against that construct was significantly greater than zero. Missing data, either a missing judgement or a missing confidence rating were coded zero. The weighted judgement of that item by that judge, therefore, was zero and was entered as such into the one sample t-test. Hochberg's correction was used to correct for multiple tests [20]. Application of the Hochberg's correction identified statistical significance was achieved with t-values that corresponded to a p value of ≤ 0.001.

#### Inter-rater reliability

Intraclass correlation coefficients (ICC) and their 95% confidence intervals (95%C.I.), were used to assess agreement between judges across all 38 items and for each construct, i.e. I, A and P judgements. The weighted judgements were used to calculate the ICC using the two-way mixed model with measure of consistency.

## Results

### Reliability of participant performance

The ICC for all judgements across all 38 items was 0.96 (95% C.I. 0.94–0.97). The ICC for each construct was as follows, 0.96 (95% C.I. 0.96–0.99) for I judgements, 0.96 (95% C.I. 0.94–0.97) for A judgements and 0.94 (95% C.I. 0.86–0.98) for P judgements. Examination of the contribution of each participant to the ICC, for all judgements and for each construct revealed all participants to be performing equally well; therefore, all 24 participants were included in the subsequent analyses.

### DCV Analyses

Thirty-four of the 38 items were classified to one or more of the ICF constructs (see Table 2). Twenty-seven items were identified as pure construct measures being related to a single ICF construct only. Five items were uniquely related to the impairment construct; all five items were from the main section of the DASH. Nineteen items were uniquely related to the activity limitations construct; 15 items from the main section of the DASH and two from each optional section. Three items were identified as pure measures of the participation restriction construct; two from the main section, one from the optional work section. Seven items were matched to both the activity limitations and participations constructs; this was the only form of mixed item within the questionnaire; five mixed items were from the main section of the questionnaire and two from the sport/music section. Judges failed to agree on the classification of 4 items; these were items 10, 29 and 30 from the main section of the questionnaire and item three from the optional work module.

**Table 2:** Classification of each DASH item

	t-values (df = 23) for the weighted judgements against the three ICF constructs			
DASH item	Impairment	Activity Limitations	Participation Restrictions	Designation
Core items				
Please rate your ability to do the following activities in the last week by circling the number below the appropriate response.				

1. Open a tight or new jar.	-3.0	16.6*	-1.1	A
2. Write.	-1.9	26.3*	0.5	A
3. Turn a key.	-2.1*^i^	10.3*	-0.7	A
4. Prepare a meal.	-4.4	8.6*	2.9	A
5. Push open a heavy door.	-2.8	36.7*	-0.9	A
6. Place an object on a shelf above your head.	-1.6	11.9*	-2.8	A
7. Do heavy household chores (e.g., wash walls, wash floors).	-3.9	8.7*	0.5	A
8. Garden or do yard work.	-4.0	4.7*	1.9	A
9. Make a bed.	-4.3*^i^	32.9*	0.2	A
10. Carry a shopping bag or briefcase.	-2.1	3.5	-0.4	NONE
11. Carry a heavy object (over 10 lbs).	-2.5	29.1*	-1.5	A
12. Change a lightbulb overhead.	-4.3*^i^	10.1*	-0.9	A
13. Wash or blow dry your hair.	-2.8	26.8*	-0.2	A
14. Wash your back.	-2.9	25.8*	-1.7	A
15. Put on a pullover sweater.	-1.8	31.8*	-2.4	A
16. Use a knife to cut food.	-2.0	26.8*	0.8	A
17. Recreational activities which require little effort (e.g., cardplaying, knitting, etc.).	-5.3*^i^	5.2*	10.5*	AP
18. Recreational activities in which you take some force or impact through your arm, shoulder or hand (e.g., golf, hammering, tennis, etc.).	-0.8	4.5*	4.8*	AP
19. Recreational activities in which you move your arm freely (e.g., playing frisbee, badminton, etc.).	-1.1	5.7*	9.6*	AP
20. Manage transportation needs (getting from one place to another).	-6.4*^i^	3.2	4.8*	P
21. Sexual activities.	-1.8	7.9*	10.3*	AP
22. During the past week, *to what extent *has your arm, shoulder or hand problem interfered with your normal social activities with family, friends, neighbours or groups?	-1.0	0.3	32.7*	P
23. During the past week, were you limited in your work or other regular daily activities as a result of your arm, shoulder or hand problem?	-1.5	7.3*	4.8*	AP
24. Arm, shoulder or hand pain.	7.0*	-9.1*^a^	-8.3*^p^	I
25. Arm, shoulder or hand pain when you performed any specific activity.	9.8*	2.1	-2.2	I
26. Tingling (pins and needles) in your arm, shoulder or hand.	5.8*	-8.9*^a^	-8.9*^p^	I
27. Weakness in your arm, shoulder or hand.	11.2*	-5.3*^a^	-6.3*^p^	I
28. Stiffness in your arm, shoulder or hand.	11.1*	-6.0*^a^	-6.8*^p^	I
29. During the past week, how much difficulty have you had sleeping because of the pain in your arm, shoulder or hand?	0.9	1.2	-0.1	NONE
30. I feel less capable, less confident or less useful because of my arm, shoulder or hand problem.	-0.6	-2.1	1.9	NONE

Work module (optional)				
Please circle the number that best describes your physical ability in the past week. Did you have any difficulty:				

1. using your usual technique for your work?	-1.1	7.2*	2.1	A
2. doing your usual work because of arm, shoulder or hand pain?	-0.5	3.6	4.3*	P
3. doing your work as well as you would like?	-5.1*^i^	3.9	3.0	NONE
4. spending your usual amount of time doing your work?	-4.6*^i^	5.1*	3.6	A

Sports/performing arts module (optional)				
Please circle the number that best describes your physical ability in the past week. Did you have any difficulty:				

1. using your usual technique for playing your instrument or sport?	-1.8	17.9*	3.2	A
2. playing your musical instrument or sport because of arm, shoulder or hand pain?	0.0	10.6*	6.2*	AP
3. playing your musical instrument or sport as well as you would like?	-3.3	5.3*	3.7	A
4. spending your usual amount of time practising or playing your instrument or sport?	-4.9*^i^	5.4*	5.6*	AP

*p < 0.001, item is a significant match to the theoretical construct; * ^i^p < 0.001, item is a significant NO match to the definition of impairment; *^a^p < 0.001, item is a significant NO match to the definition of activity limitations; *^p^p < 0.001, item is a significant NO match to the definition of participation restrictions. NONE = items for which there was no significant agreement between the judges.

## Discussion

The DASH contained 27 pure construct measures and can, therefore, be used to measure each ICF construct without measurement confound.

A recently published study used 4 judges to link the ICF to the DASH items [21]. In this study the ICF coding taxonomy was employed and individual DASH items were assigned one or more ICF code. This strategy enables the identification of I items but does not enable A to be distinguished from P because the ICF coding taxonomy fails to discriminate between these two theoretically distinct constructs. Nonetheless, these findings were consistent with the DCV analysis; with the exception of one item, all items identified as measuring I in the current study were assigned to ICF codes within the body functions category in the linkage study. The exception was item 29 which was not classified in the current study due to a lack of agreement between judges; in the Drummond et al study item 29, which concerns sleeping difficulties, received a body function coding. The I items within the DASH primarily focus on pain and muscle stiffness or weakness, previous studies have identified pain items as measures of the impairment construct [13].

All other items (except item 30) were coded as "activities **and **participation" by Drummond et al. In the current study these items were also coded as A or P or AP. However, the current data enables A and P items to be distinguished and enables mixed items to be clearly identified. This has the advantage of enabling the discriminant measurement of each type of outcome.

The availability of discriminant outcome measures is especially important in assessing outcomes in intervention studies. Being able to assess the impact of an intervention on I, A and P outcomes, in a manner that is free of measurement confound, provides the means to assess the success of an intervention within each outcome domain. For example, in the case of wrist fracture, x-ray may indicate the fracture (I) to be healed but the patient may not have regained full function (A) and may remain afraid of going outside (P). Measurement of outcome in this patient would be over positive if only the impairment outcome were measured. The DASH produces a single score; consequently it does not distinguish between the three ICF health outcomes as it is currently used. However, its content is capable of producing an impairment score; an activity limitations score and a participation restriction score as well as the standard single score. The standard method of scoring the DASH generates a simple mean score across all items and converts this mean score to a 0–100 scale. The standard method of scoring the questionnaire could be applied to the items measuring each health outcome, such that, a respondent would generate a score between 0–100 for each health outcome [see Additional files 1 and 2]. We do not advocate the standard method of scoring the DASH be replaced. Rather, the availability of the DCV analysis of the DASH for clinicians and researchers enables the instrument to be individualised to the requirements of a given situation, be that the particular needs of an individual patient or the requirements of a specific research question. Consequently, it enables the existing evidence base to be developed cumulatively.

## Conclusion

This study adds to previous research in identifying pure measurement items for each of the three ICF outcomes. It provides a means to generate three distinct outcome measures for arm, shoulder and hand trauma, namely impairment, activity limitations and participation restrictions. As a consequence, the impact of interventions, such as surgery, on each health outcome can be identified.

## Competing interests

The authors declare that they have no competing interests.

## Authors' contributions

DD collected and analysed the data and is the principle author of the paper. MJ, MMcQ and CC-B provided a critical review of the manuscript. All authors read and approved the final manuscript.

## Pre-publication history

The pre-publication history for this paper can be accessed here:



## Supplementary Material

Additional file 1 DASH ICF SYNTAX. Syntax for use with SPSS analysis software. This syntax will calculate pure I, A and P scores and a mixed AP score from the DASH questionnaire, measured at up to two time points. The syntax should be applied to an SPSS data file formatted as described in additional file 2. Click here for file

Additional file 2 DASH ICF SPSS datafile. An empty SPSS data file formatted to support the syntax provided in additional file 1. Click here for file
