# Supplementary material for: The Disabilities of the Arm, Shoulder and Hand Questionnaire (DASH) can measure the impairment, activity limitations and participation restriction constructs from the International Classification of Functioning, Disability and Health (ICF)
Source: BMC Musculoskelet Disord. 2008 Aug 20;9:114. doi: 10.1186/1471-2474-9-114 (PMC2533660; doi:10.1186/1471-2474-9-114)
Supplement: Additional file 1 — DASH ICF SYNTAX. Syntax for use with SPSS analysis software. This syntax will calculate pure I, A and P scores and a mixed AP score from the DASH questionnaire, measured at up to two time points. The syntax should be applied to an SPSS data file formatted as described in additional file 2. [file 1471-2474-9-114-S1.pdf]

```
*Single time point measurement.

*IMPAIRMENT SCORE CALCULATION.
RECODE
  pain activitypain tingling weakness stiffness  (SYSMIS=0)  .
EXECUTE .
COUNT
  ICOUNT = pain activitypain tingling weakness stiffness  (1)  pain
  activitypain tingling weakness stiffness  (2)  pain activitypain tingling
  weakness stiffness  (3)  pain activitypain tingling weakness stiffness  (4)
  pain activitypain tingling weakness stiffness  (5)  .
VARIABLE LABELS ICOUNT 'NUMBER OF IMPAIRMENT ITEMS COMPLETED' .
EXECUTE .
COMPUTE IAVERAGE = (pain + activitypain + tingling + weakness + stiffness )
 /ICOUNT .
VARIABLE LABELS IAVERAGE 'AVERAGE I SCORE'
EXECUTE .
COMPUTE IMPAIRMENT = (IAVERAGE-1) * 25.
VARIABLE LABELS IMPAIRMENT 'IMPAIRMENT SCORE OUT OF 100'
EXECUTE.

*ACTIVITY LIMITATIONS SCORE CALCULATION.
RECODE
  jar write key meal door shelf chores yard bed object lightbulb hair wash
  sweater knife  (SYSMIS=0)  .
EXECUTE .
COUNT
  ACOUNT = jar write key meal door shelf chores yard bed object lightbulb
  hair wash sweater knife  (1)  jar write key meal door shelf chores yard bed
  object lightbulb hair wash sweater knife  (2)  jar write key meal door shelf
  chores yard bed object lightbulb hair wash sweater knife  (3)  jar write key
  meal door shelf chores yard bed object lightbulb hair wash sweater knife
  (4)  jar write key meal door shelf chores yard bed object lightbulb hair
  wash sweater knife  (5)  .
VARIABLE LABELS ACOUNT 'NUMBER OF ACTIVITY LIMITATIONS  ITEMS COMPLETED' .
EXECUTE .
COMPUTE AAVERAGE = (jar + write + key + meal + door + shelf + chores + yard + bed + object +
lightbulb +
  hair + wash + sweater + knife)/ACOUNT.
VARIABLE LABELS AAVERAGE 'AVERAGE A SCORE'
EXECUTE.
COMPUTE ACTIVITYLIM = (AAVERAGE-1) * 25.
VARIABLE LABELS ACTIVITYLIM  'ACTIVITY LIMITATIONS SCORE OUT OF 100'
EXECUTE.

*PARTICIPATION RESTRICTION SCORE CALCULATION.
RECODE
```

```
  transport social  (SYSMIS=0)  .
EXECUTE .
COUNT
  PCOUNT = transport social  (1)  transport social  (2)  transport
  social  (3)  transport social  (4)  transport social  (5)  .
VARIABLE LABELS PCOUNT 'NUMBER OF PARTICIPATION RESTRICTIONS ITEMS COMPLETED' .
EXECUTE .
COMPUTE PAVERAGE = (transport + social)/PCOUNT .
VARIABLE LABELS PAVERAGE 'AVERAGE P SCORE'
EXECUTE .
COMPUTE PARTRESTRICT = (PAVERAGE-1) * 25.
VARIABLE LABELS PARTRESTRICT  'PARTICIPATIONS RESTRICTIONS SCORE OUT OF 100'
EXECUTE.

*ACTIVITY LIMITATIONS AND PARTICIPATION RESTRICTIONS MIXED ITEMS CALCULATION.
RECODE
  sex knitting tennis badminton daily  (SYSMIS=0)  .
EXECUTE .
COUNT
  APCOUNT = sex knitting tennis badminton daily  (1)  sex knitting tennis badminton
  daily  (2)  sex knitting tennis badminton daily  (3)  sex knitting tennis badminton
  daily  (4)  sex knitting tennis badminton daily  (5)  .
VARIABLE LABELS APCOUNT 'NUMBER OF A AND P MIXED ITEMS COMPLETED' .
EXECUTE .
COMPUTE APAVERAGE = (sex + knitting + tennis + badminton + daily)/APCOUNT .
VARIABLE LABELS APAVERAGE 'AVERAGE AP SCORE'
EXECUTE .
COMPUTE APMIXED = (APAVERAGE-1) * 25.
VARIABLE LABELS APMIXED  'A AND P MIXED SCORE OUT OF 100'
EXECUTE.


*Time 2 measurement.

*IMPAIRMENT SCORE CALCULATION Time 2.
RECODE
  pain2 activitypain2 tingling2 weakness2 stiffness2  (SYSMIS=0)  .
EXECUTE .
COUNT
  IT2COUNT = pain2 activitypain2 tingling2 weakness2 stiffness2  (1)  pain2 activitypain2 tingling2
weakness2 stiffness2  (2)
  pain2 activitypain2 tingling2 weakness2 stiffness2  (3)  pain2 activitypain2 tingling2 weakness2
stiffness2  (4)
  pain2 activitypain2 tingling2 weakness2 stiffness2  (5)  .
VARIABLE LABELS IT2COUNT 'NUMBER OF IMPAIRMENT ITEMS COMPLETED AT TIME 2' .
EXECUTE .
COMPUTE IT2AVERAGE = (pain2 + activitypain2 + tingling2 + weakness2 + stiffness2 )
 /IT2COUNT .
VARIABLE LABELS IT2AVERAGE 'AVERAGE I SCORE AT TIME 2'
EXECUTE .
COMPUTE IMPAIRMENTT2 = (IT2AVERAGE-1) * 25.
```

```
VARIABLE LABELS IMPAIRMENTT2 'IMPAIRMENT SCORE OUT OF 100 AT TIME 2'
EXECUTE.




*ACTIVITY LIMITATIONS SCORE CALCULATION.
RECODE
  jar2 write2 key2 meal2 door2 shelf2 chores2 yard2 bed2 object2 lightbulb2 hair2 wash2
  sweater2 knife2  (SYSMIS=0)  .
EXECUTE .
COUNT
  AT2COUNT = jar2 write2 key2 meal2 door2 shelf2 chores2 yard2 bed2 object2 lightbulb2 hair2 wash2
  sweater2 knife2  (1)  jar2 write2 key2 meal2 door2 shelf2 chores2 yard2 bed2 object2 lightbulb2 hair2
wash2
  sweater2 knife2  (2)  jar2 write2 key2 meal2 door2 shelf2 chores2 yard2 bed2 object2 lightbulb2 hair2
wash2
  sweater2 knife2  (3)  jar2 write2 key2 meal2 door2 shelf2 chores2 yard2 bed2 object2 lightbulb2 hair2
wash2
  sweater2 knife2   (4)  jar2 write2 key2 meal2 door2 shelf2 chores2 yard2 bed2 object2 lightbulb2 hair2
wash2
  sweater2 knife2  (5)  .
VARIABLE LABELS AT2COUNT 'NUMBER OF ACTIVITY LIMITATIONS  ITEMS COMPLETED AT TIME
2' .
EXECUTE .
COMPUTE AT2AVERAGE = (jar2 + write2 + key2 + meal2 + door2 + shelf2 + chores2 + yard2 + bed2 +
object2 + lightbulb2 +
  hair2 + wash2 + sweater2 + knife2)/AT2COUNT.
VARIABLE LABELS AT2AVERAGE 'AVERAGE A SCORE AT TIME 2'
EXECUTE.
COMPUTE ACTIVITYLIMT2 = (AT2AVERAGE-1) * 25.
VARIABLE LABELS ACTIVITYLIMT2  'ACTIVITY LIMITATIONS SCORE OUT OF 100 AT TIME 2'
EXECUTE.


*PARTICIPATION RESTRICTION SCORE CALCULATION.
RECODE
  transport2  social2  (SYSMIS=0)  .
EXECUTE .
COUNT
  PT2COUNT = transport2  social2  (1)  transport2  social2  (2)  transport2
   social2  (3)  transport2 social2  (4)  transport2 social2  (5)  .
VARIABLE LABELS PT2COUNT 'NUMBER OF PARTICIPATION RESTRICTIONS ITEMS COMPLETED
AT TIME 2' .
EXECUTE .
COMPUTE PT2AVERAGE = (transport2 + social2)/PT2COUNT .
VARIABLE LABELS PT2AVERAGE 'AVERAGE P SCORE AT TIME 2'
EXECUTE .
COMPUTE PARTRESTRICTT2 = (PT2AVERAGE-1) * 25.
VARIABLE LABELS PARTRESTRICTT2  'PARTICIPATIONS RESTRICTIONS SCORE OUT OF 100 AT
TIME 2'
```

```
EXECUTE.

*ACTIVITY LIMITATIONS AND PARTICIPATION RESTRICTIONS MIXED ITEMS CALCULATION.
RECODE
 sex2 knitting2 tennis2 badminton2 daily2  (SYSMIS=0)  .
EXECUTE .
COUNT
  APT2COUNT = sex2 knitting2 tennis2 badminton2 daily2  (1)  sex2 knitting2 tennis2 badminton2 daily2
(2)  sex2 knitting2 tennis2 badminton2 daily2  (3)  sex2 knitting2 tennis2 badminton2 daily2  (4)  sex2
knitting2 tennis2 badminton2 daily2  (5)  .
VARIABLE LABELS APT2COUNT 'NUMBER OF A AND P MIXED ITEMS COMPLETED AT TIME 2' .
EXECUTE .
COMPUTE APT2AVERAGE = (sex2 + knitting2 + tennis2 + badminton2 + daily2)/APT2COUNT .
VARIABLE LABELS APT2AVERAGE 'AVERAGE AP SCORE AT TIME 2'
EXECUTE .
COMPUTE APT2MIXED = (APT2AVERAGE-1) * 25.
VARIABLE LABELS APT2MIXED  'A AND P MIXED SCORE OUT OF 100 AT TIME 2'
EXECUTE.
```
