# Supplementary material for: The Disabilities of the Arm, Shoulder and Hand Questionnaire (DASH) can measure the impairment, activity limitations and participation restriction constructs from the International Classification of Functioning, Disability and Health (ICF)
Source: BMC Musculoskelet Disord. 2008 Aug 20;9:114. doi: 10.1186/1471-2474-9-114 (PMC2533660; doi:10.1186/1471-2474-9-114)
Supplement: Additional file 2 — DASH ICF SPSS datafile. An empty SPSS data file formatted to support the syntax provided in additional file 1. [file 1471-2474-9-114-S2.pdf]

	jar	write	key	meal	door	shelf	chores	yard	bed	briefcase	object	lightbulb
1	.	.	.	.	.	.	.	.	.	.	.	.
2	.	.	.	.	.	.	.	.	.	.	.	.
3	.	.	.	.	.	.	.	.	.	.	.	.
4	.	.	.	.	.	.	.	.	.	.	.	.
5	.	.	.	.	.	.	.	.	.	.	.	.
6	.	.	.	.	.	.	.	.	.	.	.	.
7	.	.	.	.	.	.	.	.	.	.	.	.
8	.	.	.	.	.	.	.	.	.	.	.	.
9	.	.	.	.	.	.	.	.	.	.	.	.
10	.	.	.	.	.	.	.	.	.	.	.	.

	hair	wash	sweater	knife	knitting	tennis	badminton	transport	sex	social	daily	pain
1	.	.	.	.	.	.	.	.	.	.	.	.
2	.	.	.	.	.	.	.	.	.	.	.	.
3	.	.	.	.	.	.	.	.	.	.	.	.
4	.	.	.	.	.	.	.	.	.	.	.	.
5	.	.	.	.	.	.	.	.	.	.	.	.
6	.	.	.	.	.	.	.	.	.	.	.	.
7	.	.	.	.	.	.	.	.	.	.	.	.
8	.	.	.	.	.	.	.	.	.	.	.	.
9	.	.	.	.	.	.	.	.	.	.	.	.
10	.	.	.	.	.	.	.	.	.	.	.	.

	activitypain	tingling	weakness	stiffness	sleeping	confidence	wtechnique	wusual	wwell	wtime	stechnique	splay
1	.	.	.	.	.	.	.	.	.	.	.	.
2	.	.	.	.	.	.	.	.	.	.	.	.
3	.	.	.	.	.	.	.	.	.	.	.	.
4	.	.	.	.	.	.	.	.	.	.	.	.
5	.	.	.	.	.	.	.	.	.	.	.	.
6	.	.	.	.	.	.	.	.	.	.	.	.
7	.	.	.	.	.	.	.	.	.	.	.	.
8	.	.	.	.	.	.	.	.	.	.	.	.
9	.	.	.	.	.	.	.	.	.	.	.	.
10	.	.	.	.	.	.	.	.	.	.	.	.

	swell	stime	BLANK	jar2	write2	key2	meal2	door2	shelf2	chores2	yard2	bed2
1	.	.		.	.	.	.	.	.	.	.	.
2	.	.		.	.	.	.	.	.	.	.	.
3	.	.		.	.	.	.	.	.	.	.	.
4	.	.		.	.	.	.	.	.	.	.	.
5	.	.		.	.	.	.	.	.	.	.	.
6	.	.		.	.	.	.	.	.	.	.	.
7	.	.		.	.	.	.	.	.	.	.	.
8	.	.		.	.	.	.	.	.	.	.	.
9	.	.		.	.	.	.	.	.	.	.	.
10	.	.		.	.	.	.	.	.	.	.	.

	briefcase2	object2	lightbulb2	hair2	wash2	sweater2	knife2	knitting2	tennis2	badminton2	transport2	sex2
1	.	.	.	.	.	.	.	.	.	.	.	.
2	.	.	.	.	.	.	.	.	.	.	.	.
3	.	.	.	.	.	.	.	.	.	.	.	.
4	.	.	.	.	.	.	.	.	.	.	.	.
5	.	.	.	.	.	.	.	.	.	.	.	.
6	.	.	.	.	.	.	.	.	.	.	.	.
7	.	.	.	.	.	.	.	.	.	.	.	.
8	.	.	.	.	.	.	.	.	.	.	.	.
9	.	.	.	.	.	.	.	.	.	.	.	.
10	.	.	.	.	.	.	.	.	.	.	.	.

	social2	daily2	pain2	activitypain	tingling2	weakness2	stiffness2	sleeping2	confidence	wtechnique	wusual2	wwell2
1	.	.	.	.	.	.	.	.	.	.	.	.
2	.	.	.	.	.	.	.	.	.	.	.	.
3	.	.	.	.	.	.	.	.	.	.	.	.
4	.	.	.	.	.	.	.	.	.	.	.	.
5	.	.	.	.	.	.	.	.	.	.	.	.
6	.	.	.	.	.	.	.	.	.	.	.	.
7	.	.	.	.	.	.	.	.	.	.	.	.
8	.	.	.	.	.	.	.	.	.	.	.	.
9	.	.	.	.	.	.	.	.	.	.	.	.
10	.	.	.	.	.	.	.	.	.	.	.	.

	wtime2	stechnique	splay2	swell2	stime2
1	.	.	.	.	.
2	.	.	.	.	.
3	.	.	.	.	.
4	.	.	.	.	.
5	.	.	.	.	.
6	.	.	.	.	.
7	.	.	.	.	.
8	.	.	.	.	.
9	.	.	.	.	.
10	.	.	.	.	.
